# Translation in cardiovascular stents and occluders: From biostable to fully degradable

**DOI:** 10.1002/btm2.10066

**Published:** 2017-07-19

**Authors:** Yingying Huang, Yee Shan Wong, Herr Cheun Anthony Ng, Freddy Y. C. Boey, Subbu Venkatraman

**Affiliations:** ^1^ School of Materials Science and Engineering Nanyang Technological University Singapore 639798 Singapore

**Keywords:** cardiovascular implants, drug‐eluting stent, fully‐bioresorbable stents, occlusion devices/occluders

## Abstract

Cardiovascular disease is a major cause of morbidity and mortality, especially in developed countries. Most academic research efforts in cardiovascular disease management focus on pharmacological interventions, or are concerned with discovering new disease markers for diagnosis and monitoring. Nonpharmacological interventions with therapeutic devices, conversely, are driven largely by novel materials and device design. Examples of such devices include coronary stents, heart valves, ventricular assist devices, and occluders for septal defects. Until recently, development of such devices remained largely with medical device companies. We trace the materials evolution story in two of these devices (stents and occluders), while also highlighting academic contributions, including our own, to the evolution story. Specifically, it addresses not only our successes, but also the challenges facing the translatability of concepts generated via academic research.

## 1 INTRODUCTION

Advances in nonpharmacological management of cardiovascular disease are largely driven by clever use of established biomaterials, or the development of new ones. When we talk of advances in management, we refer to patient benefits such as reducing the complexity of the procedures used, shorter healing periods and arresting the progression of disease. The complexities and morbidities associated with open heart surgery are well‐known. Any advance that results in minimally invasive deployment is of substantial benefit to the patients and for the healthcare system as a whole. This is significant since management of cardiovascular disease is the primary healthcare cost for the developed world. Pharmacological intervention in post‐Myocardial Infarction (post‐MI) patients deals predominantly with blood‐pressure management and anticlotting medication that ironically is necessitated by the presence of the blood‐contacting device (stent, in this case); the search for the elusive “plaque‐buster” continues but seems to have lost momentum in recent times. Thus, in cardiology, implanted devices dominate in disease management, and in this paper, we review advances in biomaterials‐based design of implanted devices, more specifically stents and occluders, including highlighting our own contributions to design and performance enhancements. The underlying theme of our review is translatability of concepts, preferably as demonstrated in the clinic or in well‐accepted animal models.

Both the design and the materials of implanted devices such as heart valves, coronary stents, and occluders have undergone dramatic changes driving the transition from surgical implantation to percutaneous deployment. This is a tremendous leap in the use of device technology, and confers obvious benefits to the patient: the procedure is minimally invasive, it is relatively painless and can be done as an outpatient procedure; and the subsequent healing period is drastically shortened. Both patient and healthcare system costs are thus dramatically reduced.

The revolution in management of MI started with the advent of balloon angioplasty in the late 1970s; this was made possible via the invention of the balloon catheter, by Dr. Gruntzig.^1^, ^2^ This catheter incorporated a remarkable segment (the “balloon”) that could be expanded in situ at the stenosed section of the coronary artery by hydrostatic pressure, while guided by X‐ray fluoroscopy to the site of blockage. Most importantly, the catheter was inserted through an artery in the leg or arm, and contained radio‐opaque segments that made it visible in X‐ray. Although balloon angioplasty replaced bypass surgery as the first option for patients with a single blocked artery, the procedure still led to re‐blockage of the stenosed artery, resulting in re‐intervention rates of 40–50% that were unacceptable.

As one of the reasons for the restenosis was vessel recoil, the insertion of a slotted stainless‐steel tube (stent) concurrent or subsequent to balloon angioplasty was approved in 1993. This concept of a coronary stent, envisaged by a cardiologist named Julio Palmas was so revolutionary that he could not find anyone willing to fund it, until a Texas restauranteur named Phil Romano put up some initial funding of $250,000. Following successful human trials this stent known as the Palmas stent was approved in 1994.^3^

From such humble beginnings, the bare metal stent has undergone significant design and material changes to the present‐day Co‐Cr stent, which has a strut thickness of about 90 microns.^4^ Ironically, while the bare metal stent was being deployed more and more widely, it was noted that restenosis rates were still around 20–30%; this restenosis was triggered largely by uninhibited growth of smooth muscle cells (SMCs) in response to the trauma caused by the stenting procedure. This led to the invention of a drug‐eluting stent (DES), a combination of pharmaceutical/device action, first developed by Johnson & Johnson and approved in 2003.^5^ For a while, DES enjoyed the lion's share of the stent market until late‐stage thrombotic events began to be noticed in long‐term studies following deployment. These thrombosis events were traced to incomplete or delayed endothelialization of the stent, caused ironically by the use of drugs such as paclitaxel and sirolimus that did not discriminate between proliferating SMCs and ECs. Although such thrombotic events were rare, they were fatal. This led a few cardiologists to think about fully resorbable stents, as evidence mounted that stents were really needed only for about 6–9 months.^6^, ^7^ After this period, stents were not only redundant but also a liability, as their presence necessitated the use of anticlotting drugs and their presence was counter‐indicated for certain procedures such as MRI imaging.

The pace of research progress in this area is astounding, and largely led by medical‐device industry R&D up to the point where the first notion of fully resorbable stents was mooted. The fully absorbable stent concept arose in a medical school, and was developed by Japanese researchers, Dr. Igaki and Tamai, and eventually licensed to Kyoto Medical Planning. This nondrug eluting stent was first reported to have undergone human trials in 2000.^8^ The Igaki–Tamai stent used a hydrolytically degradable polymer called Poly (l‐lactide) which had been used as a suture material in surgery, and is known to degrade over a period of 12–24 months.

Our involvement in the fully degradable stent research started in 2003, with a grant from Singapore's Ministry of Education. Concurrently, we also worked on a metal stent coated with a biodegradable polymer that delivered two drugs, an antiproliferative (sirolimus) and antithrombotic (triflusal).^9^ We describe our efforts to translate these concepts to the clinic in Sections 2.2.1 and 3.1.1. All abbreviations used are listed in Table 5.

## 2 DUAL‐DRUG‐ELUTING STENTs

### 2.1 Background on drug types

The first generation of DES includes the sirolimus‐eluting stent (Cypher®) and the paclitaxel‐eluting stent (Taxus®), with both demonstrating impressive reductions in restenosis from 50 to 20/30% compared with bare metal stents (BMS). However, due to incomplete healing, the incidence of late stent thrombosis (LST) marred their long‐term use, especially after discontinuation of dual antiplatelet therapy.^5^

Paclitaxel is a lipophilic molecule with potent antiproliferative and antimigratory activities. The drug is a microtubule‐stabilizing agent, which enhances formation of microtubular polymerized structures and thus, decreases the concentration of tubulin required for new microtubule formation. Paclitaxel affects primarily the M phase of the cell cycle inhibiting growth factor‐induced DNA synthesis and cell proliferation, and leads to apoptosis or cell death. Concerns regarding cardiotoxicity of paclitaxel has been one of the reasons for its slow eclipse.

Currently, it appears that the limus‐family of eluting stents dominates the market, as shown in Table 1. Six limus family compounds have been used in DES^10^: these compounds target either the mammalian target of rapamycin (mTOR) (sirolimus, everolimus, zotarolimus, and biolimus A9) or calcineurin (tacrolimus and pimecrolimus). The mTOR inhibitors (sirolimus, everolimus, zotarolimus, and biolimus A9) share an almost identical lipophilic chemical structure and bind to their major cytosolic FK‐506 binding protein‐12 (FKBP12), forming a complex which subsequently inhibits the mTOR. The major cellular effects include a decrease of the positive (blockage of the p70S6 kinase pathway of the cyclin‐dependent kinases) and an increase of the negative (through inhibitor p27 kip1) regulators of the cell cycle^11^; they stop the cell cycle at the G0/G1 phase inhibiting both cell (mainly smooth muscle cells) proliferation and migration, so the mechanism of action is cytostatic rather than cytotoxic. Tacrolimus and pimecrolimus are not analogs of the archetypal rapamycin; after they bind intracellularly to FKBP12, the complex in turn binds to and blocks calcineurin, and in this way inhibits the T‐cell transduction pathways and the synthesis of pro‐inflammatory cytokines.^12^ In vitro cell work indicates that tacrolimus allows earlier endothelial regeneration than sirolimus; however, inhibitory activity on human vascular SMCs with tacrolimus is much less than sirolimus.^13^ In this context, both everolimus‐eluting stents (EES) and biolimus‐eluting stents (BES) are the front‐runners, and likely to be approved (or already approved) as noninferior products.

**Table 1:** Limus‐family drug‐eluting stent in market

Drug(s)	Stent name (manufacturer)	Polymer(s)	Stent platform	Descriptions (drug release)	Status
Sirolimus (140 µg/cm^2^)	Cypher^TM^ (Cordis)	polyethylene‐co‐vinyl acetate (PEVA) and poly *n*‐butyl methacrylate(PBMA)	SS	80% in 30 days, and completed release at 90 days	FDA approved
Sirolimus (6.6 µg/mm)	Supralimus (Sahajanand Medical)	PLLA‐PLGA‐PCL‐PVP	SS	100% in 48 days	CE approved
Sirolimus (125 µg/cm^2^)	BioMime (Meril Life Science)	PLLA + PLGA	Co‐Cr	100% in 30 days	CE approved, Phase III trial completed by Dec 2017
Sirolimus (140 µg/cm^2^)	Orsiro (Biotronik)	PLLA + Silicon carbide layer	Co‐Cr	50% in 30 days	CE approved
Zotarolimus (10 µg/mm)	Endeavor (Medtronic)	Phosphorylcholine (PC)	Co‐Cr	95% in 15 days	CE approved**, FDA approved**
Zotarolimus (10 µg/mm)	ZoMaxx ZES (Abbott Vascular)	PC drug layer and PC topcoat	SS–tantalum	90% in 30 days	Abbott drops ZoMaxx
Zotarolimus (10 µg/mm)	Resolute (Medtronic)	BioLinx (hydrophobic C10, hydrophilic C19 and polyvinyl pyrrolidone(PVP))	Co‐Cr	85% in 60 days, and completed release at 180 days	FDA‐approved for patients with diabetes, CE approved
Everolimus (100 µg/cm^2^)	XIENCE V (Boston Scientific)	Polyvinylidene fluoride co‐ hexafluoropropylene and poly‐*n*‐butylmethacrylate	Co‐Cr	71% in 28 days, and completed release at 120 days	FDA approved
Everolimus (100 µg/cm^2^)	PROMUS PREMIER (Boston Scientific)	Polyvinylidene fluoride co‐ hexafluoropropylene and poly‐*n*‐butylmethacrylate	Pt‐Cr	71% in 28 days, and completed release at 120 days	FDA approved
Everolimus (5.6 µg/mm and 2.8 µg/mm)	SYNERGY (Boston Scientific)	PLGA	Pt‐Cr	50% in 60 days	CE approved
Novolimus (4.6 µg/mm)	DESyne BD (Elixir Medical)	PLA	Co‐Cr	90% in 90 days	CE approved
Biolimus A9 (15.6 µg/mm)	BioMatrix Flex (Biosensors)	PLA	SS	45% in 30 days	CE approved
Biolimus A9 (15.6 µg/mm)	Nobori (Terumo)	PLA	SS	45% in 30 days	CE approved
Biolimus A9 (15.6 µg/mm)	Axxess (Biosensors)	PLA	Nitinol	45% in 30 days	CE approved

### 2.2 Drug‐eluting stents in research

Long term follow‐up studies of DES have shown increased incidence of (sometimes fatal) stent thrombosis probably due to delayed endothelialization by the currently used drugs or delayed hypersensitivity reaction caused by the durable polymer; or by degraded polymer products currently used in DES.^14^, ^15^, ^16^ Researchers have been hunting for selective drugs that inhibit SMCs without affecting ECs, as well as developing dual‐drug‐eluting stent (DDES). However, these efforts have not met with much success until recently.

#### 2.2.1 Stents eluting a selective antiproliferative peptide

Lack of selectivity in inhibition has been a major drawback of current synthetic drugs used in the DES. The presence of these immunosuppressive and cytotoxic drugs invariably also delays endothelial healing of the vessel,^17^, ^18^, ^19^ which gives rise to LST. Researchers have been scouting for “selective drugs.”

Researchers at Mayo Clinic reported on a chimeric peptide termed CD‐NP or cenderitide that has interesting vasodilating properties without renal effects.^20^ Our collaborative efforts resulted in an observation that released CD‐NP inhibits human coronary artery smooth muscle cells proliferation but did not hamper human umbilical vein endothelial cells proliferation in vitro,^21^ which is not surprising given their endothelial cell origins. Based on this in vitro work, we designed a peptide‐eluting stent (or cenderitide‐eluting stent, CES) that could deliver cenderitide in a controlled fashion from a Co‐Cr stent coating matrix composed substantially of biodegradable poly(Ɛ‐caprolactone) (PCL). An array of slow, moderate and fast release profiles were attained from the addition of poly(ethylene glycol) and its copolymers in formulations for 30 days.^21^

**Figure 1:**
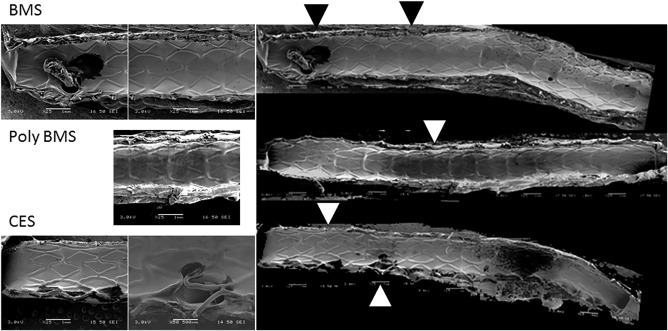
SEM image of endothelial coverage comparison^22^

Following this, a 4‐week pig study was carried out with a total of 32 stents implanted into 16 pigs.^22^ The in vivo results demonstrated significantly higher plasma levels of CD‐NP in the CES, and greater endothelial coverage of the stent struts compared with a control group (BMS and polymer‐coated stent) at poststenting, as shown in Figure 1. We believe that ensuring unperturbed endothelium regeneration is crucial to avert further progression of in‐stent restenosis and LST.

However, the CES group was not able to show a significantly different outcome in terms on stenosis, when compared to BMS. This led us to believe that 2 μg/day over 28 days dosing of CD‐NP used in this study was not sufficient to activate sufficient cGMP locally or systemically to have a significant effect on SMC proliferation. Further detailed studies, incorporating higher doses and frequent sampling, are therefore warranted.

#### 2.2.2 Dual drug‐eluting stents

Drug‐eluting stents could include two therapeutic agents, antiproliferative and pro‐healing, which would help in further enhancing the antirestenotic performance of currently available DES, and would promote healing performance. The selection of an appropriate therapeutic combination and the regulation of their release kinetics are important for successful performance of a DDES. In our review in 2014,^10^ we have surveyed and categorized DDES into three types: antiproliferative with antithrombotic, antiproliferative with re‐endothelialization promoter, and dual antiproliferative agents (independent pathways). Currently, although several DDES are in clinical trials, none has been approved for commercial use. We have developed a DDES that elutes sirolimus (an antiproliferative) and triflusal (an antithrombotic) to treat both restenosis and thrombosis.^9^ This DDES consists of a cobalt‐chromium stent with strut dimensions 0.075 × 0.080 µm (W × T), and a two‐layered coating including a biodegradable poly (lactic‐co‐glycolic) acid (PLGA) drug layer and a drug‐free top layer to control the drug release. The cumulative release profile showed an initial burst of about 30% for sirolimus, and subsequent release to 60% by day 14, and more than 70% cumulative sirolimus release after 30 days. In contrast, approximately 60% of burst release and almost complete release in about 5 days was registered for trifusal.

**Figure 2:**
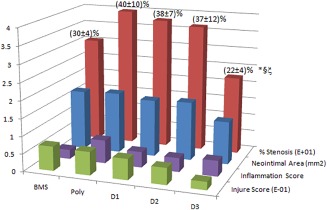
The histological results of DDES in pigs for 28 days^9^

A 24‐pig study^23^ (expanded stent/artery = 1.1:1) showed a significant reduction in restenosis after DDES implantation, compared with SES and BMS (DDES 22 ± 4% vs. SES 37 ± 12%, *p* < .036; vs. BMS 30 ± 4%, *p* < .034) at week 4, as Figure 2. We attributed the significant restenosis reduction to the prevention of thrombus formation in the early stage due to the early burst release of triflusal. It is accepted that the thrombus formed initially (1–3 days) acts as a scaffold for neointimal proliferation of SMCs, and enhances restenotic events.

This is an example of a translatable concept that has shown very promising preclinical results in an accepted animal model for restenosis. However, the generation of the strong efficacy data was reported at a time when the interventional cardiology community was turning away from DES toward BMS, because of the concerns raised regarding late‐stage thrombosis in DES‐stented patients. Hence the work went no further, due to lack of interest from the investment community. Meanwhile, the momentum regarding fully bioabsorbable stents had taken hold, and efforts were re‐directed to the more challenging but potentially more rewarding area of resorbable stents.

## 3 FULLY BIORESORBABLE STENTS

### 3.1 Fully bioresorbable stents in the market

It has been recognized for some time, that a fully resorbable stent is a preferred option for unblocking coronary arteries, deployed via percutaneous coronary intervention (PCI). The concept of a temporary multifunctional stent that enables revascularisation of the artery via a mechanical support like a metallic stent; prevention of restenosis by sustained‐delivery of an antirestonotic like a DES; and allowing the restoration of normal vasomotion after resorption, has been considered the “holy‐grail” of a fourth‐generation stenting technology.^24^ However, despite the many positive attributes that bioresorbable stents are expected to bring, there has been so far only two companies obtaining regulatory approval for such implants.

The world's first bioresorbable stent that found its way into humans was the implanted Igaki–Tamai stent at the turn of this century.^8^ The stent was made with poly‐l‐lactide (PLLA) and expanded in the artery via a heated delivery balloon. The first‐in‐human (FIM) study involved a total of 25 such stents implanted in 15 patients. Together with a second trial, clinical results were promising, but usage was not extended to PCI due to the complexity involving the thermal balloon.^25^ Although the stent was deployed percutaneously in the study, it needed to be heated to about 70°C briefly, in order to overcome the natural recoil of the viscoelastic PLLA on balloon expansion. This problem in deployment got us interested in using shape memory concepts in deploying the sent.^26^

The leader in the field of fully absorbable coronary stents is Abbott Vascular, with their Absorb BVS (Bio‐absorbable Vascular Scaffold) GT1 stent, which was approved by the U.S. FDA in July 2016, after a protracted set of clinical trials, including one (ABSORB II) in which a head‐to‐head comparison was made with the Xience Prime stent (NCT01425281). This stent received the CE Mark in December 2010,^27^, ^28^ while the Elixir stent (the DESolve platform), which is a novolimus‐eluting fully degradable stent, won the CE mark in May 2013. The success of these companies is a testament to the fact that the concept of a fully absorbable stent is now acceptable to the cardiologists, with the added benefit of stopping the dual‐antiplatelet therapy for stented patients, much sooner.

#### 3.1.1 The amaranth story: An example of academic translation

**Figure 3:**
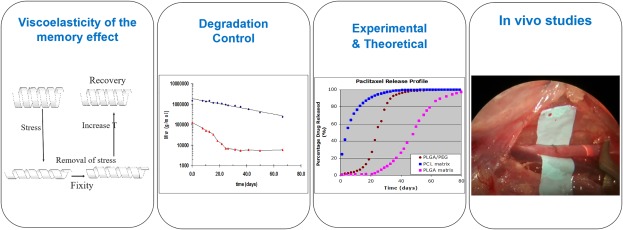
NTU Studies of biodegradable stent^26^, ^31^, ^32^

Our early efforts in the area of bioabsorbable stents were funded by Singapore's Ministry of Education. Our concept of a percutaneously deployable fully bioabsorbable stent was based on self‐expansion built into a multilayered construct.^29^ The radial strength of these constructs was studied and reported.^30^ Other studies included biodegradation, drug release from coatings, and the viscoelasticity and shape memory properties of the materials used, as shown in Figure 3.

Our initial focus was on ureteric stents, where the ureter was also subject to stenosis due to various causes; a fully bioabsorbable stent had to degrade in about 4–6 weeks in this case.^33^ Based on this work, funding was obtained from a Singaporean venture firm (Bio1 Ventures) in 2006, leading to a company called Amaranth Medical. Subsequent investment by a U.S. VC firm then redirected the technology to cardiovascular stents, a more challenging but also more lucrative market. The technology of multilayered stents was licensed from Nanyang Technological University, by Amaranth Medical in 2006.

**Figure 4:**
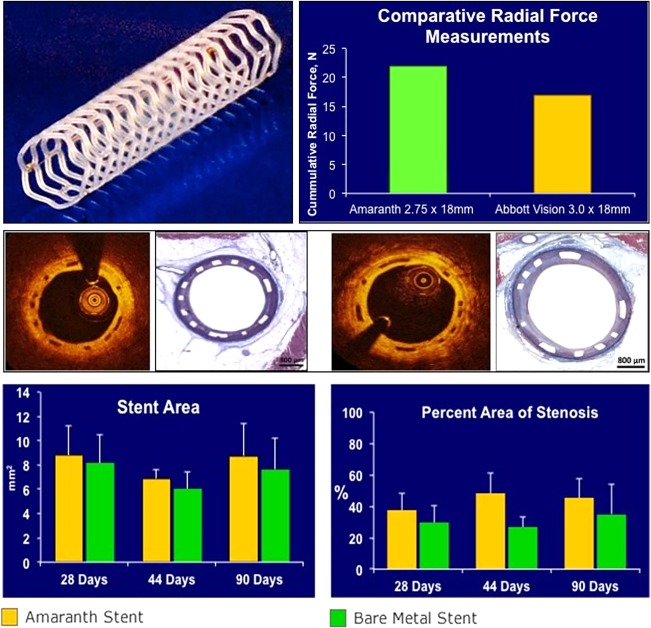
The results of Amaranth stent^34^

Further development, carried out by Amaranth led to several patented refinements including the process of making and sterilizing the stents.^29^ The first‐generation stent was named FORTITUDE™. It exhibited excellent mechanical properties, comparable to or better than the Abbott BVS stent VISION^TM^, which was ahead in terms of clinical results (Figure 4). As explained in a European PCR presentation in 2017,^35^ the FORTITUDE stent used a specially made ultrahigh molar mass PLLA of very low crystallinity, which resulted in a tougher stent that showed very few balloon‐expansion‐related fractures.

**Figure 5:**
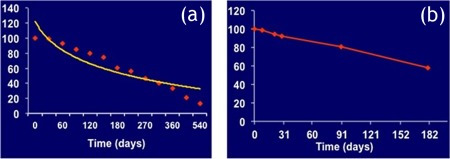
The polymer has a half‐life in vivo of about 6 months: (a) molecular weight loss in vitro and (b) molecular weight loss in vivo

In terms of degradation, the special PLLA polymer (custom‐synthesized for Amaranth) behaves as shown in the Figure 5 below:

The FORTITUDE sirolimus‐eluting bioresorbable scaffold (BRS) is currently being evaluated in two multicenter trials in centers in Colombia, South America (MEND‐II trial) and Italy (RENASCENT trial). The patients who have enrolled in these studies have symptomatic coronary artery disease and are undergoing PCI with single coronary lesions. Results from this clinical program will support a planned application for CE Mark.^34^

The second‐generation Amaranth stent is APTITUDE^TM^,^36^ which has a strut thickness of 115 microns, and a further third‐generation stent (MAGNITUDE^TM^)^37^ with a strut thickness that is sub‐100 microns, is in development and in pre‐clinical trials. U.S. FDA Approval is anticipated in 2018.

### 3.2 Fully bioresorbable stents/scaffolds in research

#### 3.2.1 Other polymer‐based scaffolds

Tyrosine‐derived polycarbonate polymers degrade into amino acids, ethanol, and carbon dioxide. An iodine‐incorporated tyrosine‐derived polycarbonate was developed by Prof Joachim Kohn's group at Rutgers State University.^38^ This fully degradable polymer has good mechanical properties, and importantly, is also visible in X‐ray fluoroscopy by virtue of the incorporated iodine. The polymer was licensed to Reva Medical (San Diego, California). The REVA stent was a nondrug eluting coronary stent implanted in an FIM trial. The study enrolled 27 patients and a high target lesion revascularization rate led to a new design, the ReZolve stent, that included sirolimus‐eluting capability.^25^ Second‐generation and third‐generation, ReZolve2, and Fantom stents, respectively, are currently in clinical trials (NCT01845311, NCT02512003, and NCT02539966) for submission for CE Marking.^10^, ^24^, ^39^

The IDEAL BioStent by Xenogenics Corporation (Woonsocket, Rhode Island) is a sirolimus‐eluting bioresorbable stent based on a polyanhydride ester mixed with salicylic acid.^25^ The stent has both antiproliferative and anti‐inflammatory properties attributable to its dual release capability for salicylic acid and sirolimus. An FIM study on an earlier version enrolled 11 patients and results were unsatisfactory due to excessive neointimal growth.^25^ The current design is under development and in clinical trials, but not much information has been released thus far. Details of these are summarized in Table 2.

**Table 2:** Fully bioresorbable coronary stents in research

Stent	Manufacturer	Material	Eluted drug	Resorption time (months)	Current status
DESolve‐2	Elixer Corporation	PLLA	Novolimus	12	CE Mark, clinical trials
Fortitude	Amaranth Medical	PLLA	–	24–36	Completed trials
Aptitude	Amaranth Medical	PLLA	Sirolimus	Unknown	Clinical trials
ART	Arterial Remodeling Technologies	PLLA/PDLLA	–	18–24	Clinical trials
FAST	Boston Scientific	PLLA	Everolimus	24–36	Clinical trials
XINSORB	Huaan Biotech	PLLA/PCL/PGA	Sirolimus	24–36	Clinical trials
MIRAGE	ManLi Cardiology	PLLA	Sirolimus	14	Clinical trials
MeRes 100	Meril Life Sciences	PLLA	Sirolimus	24	Clinical trials
Firesorb	MicroPort	PLLA	Rapamycin (sirolimus)	Unknown	Clinical trials
ReZolve2	REVA Medical	Polytyrosine‐derived polycarbonate	Sirolimus	48	Clinical trials
Fantom	REVA Medical	Deaminotyrosine‐derived polycarbonate	Sirolimus	36	Clinical trials
IDEAL BioStent	Xenogenics Corporation	Polyanhydride ester with salicylic acid	Sirolimus, salicylic acid	6–9	Clinical trials

## 4 OCCLUSION DEVICES FOR STRUCTURAL HEART DISEASE

### 4.1 Review of occlusion devices in the market

Atrial septal defect (ASD) and patent ductus arteriosus (PDA) are two major types of congenital heart diseases. ASD is defined as a persistent communication between the right and left atria. The incidence of ASD is about 1 in 2000 newborns^40^ and this accounts for 7–10% of all congenital heart defects in adults.^41^ Patients with unrepaired ASD can lead to complications such as atrial arrhythmias, pulmonary hypertension, and cardiac failure.^42^ On the contrary, PDA is an open channel allowing mixing of oxygenated and deoxygenated blood from the aorta to the pulmonary artery. The incidence of PDA is about 1 in 2000 newborns, accounting for 5–10% of all coronary heart diseases.^43^ If the PDA is large and left untreated, this condition can cause symptoms such as fatigue, difficult or rapid breathing, failure to grow normally, or eventually heart failure and death.

As an alternative to open‐heart surgery, percutaneous transcather closure of ASD has become the preferred management strategy. At present, two devices have been approved by U.S. Food and Drug Administration (FDA) through the premarket approval process for ASD closure: the Amplatzer^TM^ Atrial Septal Occluder (St. Jude Medical, Minneapolis, MN) and the GORE HELEXTM Septal Occluder (W.L. Gore & Associates, Newark, DE). In 2002, the Amplatzer Atrial Septal Occluder (ASO) was approved for the occlusion of ASDs in the secundum position, followed by FDA approval of the GORE HELEX Septal Occluder (HSO) in August 2006. The Amplatzer ASO device is a self‐expanding, repositionable, double‐disk device comprised of nitinol metal braid. Eighteen device sizes are available ranging from 4 to 38 mm corresponding to the device waist size. The left atrial disk is slightly larger than the right atrial disk, and polyester patches are sewn into the disks and waist to promote defect closure and tissue in‐growth. As of January 2012, total sales of the Amplatzer ASO device were 223,965 devices, with 72,566 devices sold in centers in the United States and the remaining 151,399 devices shipped internationally. In term of safety performance, 98.5% closure rate with no signature residual shunt (less than 2 mm) is reported at 1‐year follow‐up.^44^ Nonetheless, serious complication of device erosion has been reported, with an estimated worldwide implant rate of 0.2–0.5%.^45^ The mechanism is thought to be compliance mismatch between the left atrial disk wire mesh and the atrial wall.

The HSO device assumes a double disk configuration following deployment of a helical nitinol wire frame covered with expanded polytetrafluoroethylene (ePTFE). A maneuver is performed to lock the two disks together prior to the release of the delivery catheter. Five device sizes, ranging from 15 to 35 mm, are available and designed to close defects that are ≤18 mm in diameter. Subsequently, the HSO device has been upgraded to GORE® Cardioform Septal Occluder (GSO), which was granted CE mark in June 2011 for the treatment of patent foramen ovale (PFO) and ASD. The GSO is modified with a flexible, retrievable, double disc configuration with a petal design made of platinum‐filled nitinol frame covered with a thin hydrophilic surface‐treated ePTFE membrane to facilitate rapid endothelialization. These features have improved the device apposition ability and tissue response while keeping its atraumatic design, low septal profile with minimal septal distortion, and long‐term biocompatibility.^46^ The success rate for deployment is high (89%) with an acceptable small residual shunt is reported with GSO.^41^ Having softer disk with less frictional forces, the efficacy was similar to the ASO with a low risk of a major adverse event, such as fracture or embolization and no erosion has been reported yet, even in deficient rim complex ASDs.^47^

Besides FDA‐approved Amplatzer ASO and GORE HSO, there are other occlusion devices in the market with CE mark approval instead such as Occlutech Figulla Septal Occluder (Occlutech GmbH., Jena, Germany), Ultrasept ASD Occluder (Cardia, Inc., Eagan, MN), CeraTM ASD Occluder (Lifetech Scientific Corporation, Shenzen, PRC). In general, these devices are similar to ASO in design with self‐expanding, recapturable, double round disk made of metallic wire mesh, and polymeric patch to facilitate endothelialiation. One unique feature of these devices are that the nitinol wire frame is coated with either titanium oxide or nitride; that is associated with less thrombosis, nickel ion elution, and improved endothelial tissue growth.^48^ The occlusion devices for ASD in the market are summarized in Table 3.

**Table 3:** Occlusion devices in the market

Product name	Manufacturer	Materials	Delivery system	Remarks	Configuration
ASD closure					
Amplatzer Atrial Septal Occluder	St. Jude Medical	Nitinol; polyester fabric	6–12F	FDA (2001)	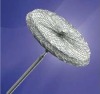
GORE HELEXTM Septal Occluder	W.L. Gore & Associates	Nitinol; ePTFE	9–13F	FDA (2006)	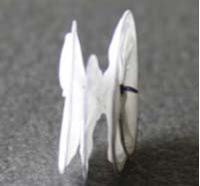
GORE® Cardioform Septal Occluder	W.L. Gore & Associates	Platinum‐filled nitinol; ePTFE with hydrophilic surface treatment	10–12F	CE Mark	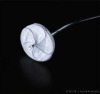
Occlutech Figulla Septal Occluder	Occlutech GmbH	Nitinol coated with titanium oxide; PET patch		CE Mark	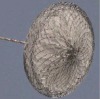
Ultrasept ASD Occluder	Cardia, Inc.	Nitinol coated with titanium nitride; polyvinyl alcohol (PVA)	9–11F	CE Mark	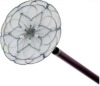
CeraTM ASD Occluder	Lifetech Scientific Corporation	Nitinol coated with titanium nitride; ePTFE	7–14F	CE Mark (2012)	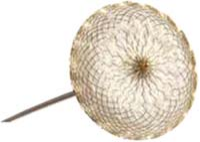
PDA closure					
Gianturco Coil	Cook Medical, Inc.	Stainless steel; Dacron fibers	3F	In market since 1976	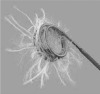
Amplatzer Duct Occluder	St. Jude Medical	Nitinol; polyester fabric	5–7F	FDA (2003)	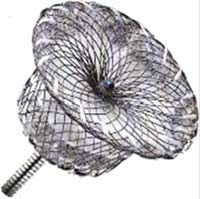
Amplatzer Duct Occluder II	St. Jude Medical	Nitinol	4–5F	FDA (2013)	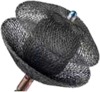
Nit‐Occlud PDA	pfm medical	Nitinol	4–5F	CE Mark (2001) FDA (2013)	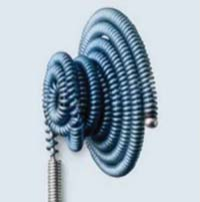
Occlutech PDA Occluder	Occlutech GmbH	Nitinol coated with titanium oxide; PET patch	6–9F	CE Mark	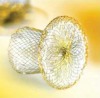
Cocoon duct occluder	Vascular Innovation Co. Ltd	Nanofusion platinum‐coated nitinol; polypropylene	6–10F	Thai FDA (2008) CE Mark (2010)	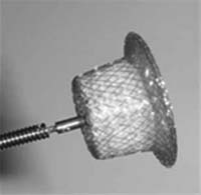
CeraTM PDA Occluder	Lifetech Scientific Corporation	Nitinol coated with titanium nitride; ePTFE		CFDA CE Mark	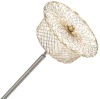
Cardio‐O‐Fix (CSO) PDA occluder	Starway Medical Technology Inc.	Nitinol; polyester fabric	7–14F	CE Mark	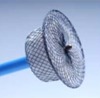

Likewise, percutaneous transcatheter closure of PDA has been developed to eliminate an extended hospital stay and convalescence associated with open surgical PDA closure. At present, FDA‐approved PDA occlusion devices include: Gianturco Coil (Cook Medical, Inc., Bloomington, IN), Amplatzer Duct Occluder (St. Jude Medical, Minneapolis, MN), and Nit‐Occlud PDA (pfm medical, Cologne, Germany). Although the Gianturco coil has never undergone formal FDA approval but it is available for clinical use for it was marketed prior to 1976 before the FDA formally acquired regulatory authority over devices. Gianturco coils are constructed of stainless steel wire interwined with Dacron fibers to induce thrombogenesis and are indicated for the closure of small (<4 mm) PDAs. Typically, one to four coils are required to completely occlude the ductus.^49^ Besides Ginaturco coil, the Nit‐Occlud PDA system is also utilized for closure of small to medium‐sized PDAs. It is similar in appearance with Ginaturco coil but has many more loops of varying diameters that stack to form a cone. Only the anterior loops of this device are positioned in the pulmonary artery while the remainder loops are generally placed in the aortic ampulla of the ductus.

For the closure of large PDAs, Amplatzer duct occluder (ADO) is the preferred choice. ADO is made of nitinol wire mesh that is configured into a cylindrical plug shape with a retention skirt to better anchor the device within the ductus and reduce embolization. Polyester fabric is sewn into the occluder to induce the thrombosis that closes the communication. In term of safety and efficacy, the successful deployment rate of ADO is 99.2% in a total of 390/393 patients with overall a 5.8% adverse event. Complete closure of the ductus was 98.4% at the 6‐month interval and 98.6% at the 12‐month interval.^50^ A second generation of ADO, Amplatzer Duct Occluder II (ADO II), was granted FDA approval on 2013. ADO II consists of fabric‐free symmetrical multilayered nitinol mesh and dual articulating discs providing high conformability to treat the nonconical ducts, small infants with larger duct diameters while achieving compete closure from an aortic or pulmonary artery deployment approach. In a series of clinical trials, it demonstrates the safety and efficacy of the ADO II to occlude PDA with diameter that is ≥2 mm with successful deployment rate of 93.3% in a total of 56/60 pediatric patients.^51^, ^52^, ^53^ The occlusion devices for PDA in the market are also summarized in Table 3.

### 4.2 Occlusion devices in preclinical research

Device closure of appropriately indicated ASD and PDA is standard of care in most countries. All current commercial devices use a metallic frame and occlusive patch material. Metallic frameworks are recognized as the major cause of serious complications such as erosion, perforation, metal fractures, nickel ions elution and allergy, reported as late as 10 years following implantation.^54^ Therefore, fully bioresorbable occluders are desirable: such an occluder not only closes the defect, but also act as a temporary scaffold for tissue regrowth and eventually “goes away” leaving host tissue behind with minimal long‐term consequences. The occlusion devices in research are summarized in Table 4.

**Table 4:** Occlusion devices in research

Product name	Manufacturer/Institute	Materials	Remarks	Current status
Biostar	NMT Medical	Nitinol; heparin‐coated, acellular, porcine‐derived collagen matrix	ASD closure; partial‐biodegradable; absorb within 2 years	Discontinued at 2011 due to closure of NMT Medical
BioTrek	NMT Medical	Poly‐4‐hydroxybutyrate	ASD closure; fully bioresorbable	Discontinued at 2011 due to closure of NMT Medical
Carag Bioresorbable Septal Occluder (CBSO)	Carag AG	PLGA monofilament framework; polyester patches	ASD/PFO closure; partial‐biodegradable; 12F delivery system	First‐in‐Human clinical trial in 2015; CE mark filed
Polycaprolactone Occlusion Device	Chang Gung University, Taiwan	PCL framework; nanofibrous PLGA/collagen membrane	ASD closure; fully bioresorbable	Bench testing^55^
ASD Occluder	Second Military Medical University, China	Polydioxanone (PDO); woven PLA; 2 tantalum particles as X‐ray markers	ASD closure; fully bioresorbable; degraded over about 24 weeks	Preclinical testing^56^
“Double Umbrella” and “Chinese Lantern” Occluder	Nanyang Technological University, Singapore	PCL; PLC; BaSO_4_	ASD/PFO closure; fully bioresorbable; complete endothelialisation at 1 month follow‐up swine study	Preclinical testing
PDA Occluder	Nanyang Technological University, Singapore	PCL; PLC; BaSO_4_	PDA closure; fully bioresorbable; complete endothelialisation at 1‐month follow‐up swine study	Preclinical testing

A partially degradable ASD closure device, Biostar, was first developed by NMT Medical and a multicenter study was conducted in 2006. The Biostar is a modification of the StarFlex device, and the Dacron covering the nitinol frames is substituted with a heparin‐coated, acellular, porcine‐derived collagen matrix that allows absorption, and replacement with human tissue (95%) within 2 years. The nitinol frame remains in the interatrial septum.^57^ Mid‐term follow‐up results at 7 months appear excellent, with deployment success rate of 97% and comparable residual shunt rates. Moreover, the BioTrek device (NMT Medical, Boston, MA) further evolved from the Biostar and is designed to be 100% bioresorbable consisting of poly‐4‐hydroxybutyrate. As of early 2010, the BioTrek was reported in preclinical testing stage.^58^ However, with the closure of NMT Medical, these studies were terminated.

**Figure 6:**
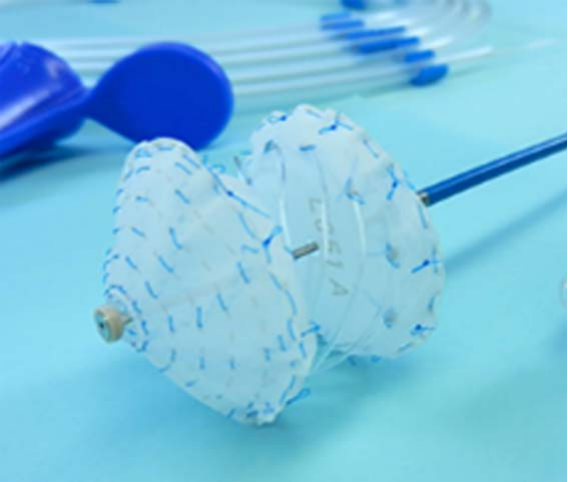
Carag Bioresorbable Septal Occluder (CBSO)^59^

Another partially degradable occluder, Carag Bioresorbable Septal Occluder (CBSO) (Carag AG, Baar, Switzerland), was recently conducted First‐in‐Human clinical trial in 2015. The CBSO consists of a PLGA monofilament framework with polyester patches attached, as depict in Figure 6. It is available in three sizes (S‐M‐L) and is designed to treat defects that are ≤25 mm in stretched diameter via the 12F sheath. An initial experience in the first‐in‐human trial, all CBSO devices were successfully implanted in seven patients (6 PFO and 1 ASD) showing complete closure in 3 out of 4 PFO patients at 6 months and complete ASD closure after 1 month.^59^

#### 4.2.1 Fully absorbable occluders at NTU

**Figure 7:**
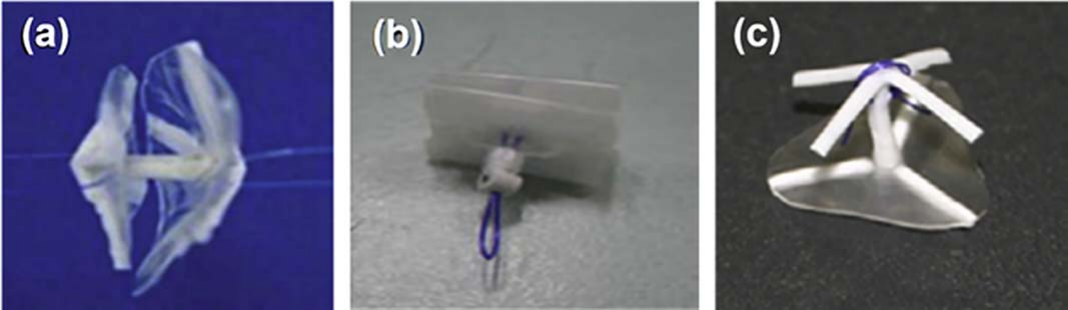
Fully bioresorbable occluders developed in our group: (a) the double umbrella; (b) the Chinese lantern and (c) PDA occluders^52^, ^56^, ^57^

At around the same time, our group also embarked on the journey of developing fully bioresorbable occluders for structural heart diseases. For ASD/PFO closure, we have developed two version of biodegradable closure devices: the “double umbrella” (2010) and the “Chinese lantern” (2011) occluders. The design of double umbrella occluder involves two discs connected with a stretchable stem, as depicted in Figure 7a. Each disc is made of four barium sulfate (BaSO4)‐doped PCL spokes covered with polylactide‐co‐ɛ‐caprolactone (PLC) film. The working principle lies in the self‐expansion of the two umbrellas linked by the stem on deployment. The preclinical study was carried out in swine model, showing that the devices were in stable position with no shunt and were well endothelialzied after the 1‐month follow‐up.^54^ The Chinese lantern occluder consists of soft portion (“head,” “waist,” and “tail” films) and structural skeleton (lock, and head tubes, and wires) with unique pull‐fold mechanism to achieve folding and sealing. On retraction of the loop wire, the head films and tail films will fold into the working structure (Figure 7b) with the waist film length being adjustable corresponding to the septum thickness. In term of material, blend of PLC, PCL, and BaSO4 were utilized for the occluder fabrication. Preclinical study was also conducted in swine model, with satisfactory X‐ray visibility. At the 1 month follow‐up, there was no shunt from right atrium to the left atrium and complete endothelialization of the device.^60^

**Table 5:** Abbreviations used in the text

Post‐myocardial infarction (post‐MI)	Poly(Ɛ‐caprolactone) (PCL)
Myocardial infarction (MI)	Poly(ethylene glycol) (PEG)
Late stent thrombosis (LST)	Poly‐L‐lactide (PLLA)
In‐stent restenosis (ISR)	Poly(lactic‐co‐glycolic) acid (PLGA)
Bare metal stents (BMS)	Polylactide‐co‐ɛ‐caprolactone (PLC)
Drug‐eluting stent (DES)	Atrial septal defect (ASD)
Dual‐drug‐eluting stents (DDESs)	Patent ductus arteriosus (PDA)
Everolimus‐eluting stents (EES)	Atrial Septal Occluder (ASO)
Biolimus‐eluting stents (BES)	HELEX Septal Occluder (HSO)
Cenderitide‐eluting stent (CES)	Expanded polytetrafluoroethylene (ePTFE)
Smooth muscle cells (SMCs)	Cardioform Septal Occluder (GSO)
Endothelial cells (ECs)	Patent foramen ovale (PFO)
Human coronary artery smooth muscle cells (HCaSMCs)	Amplatzer duct occluder (ADO)
Human umbilical vein endothelial cells (HUVECs)	Carag Bioresorbable Septal Occluder (CBSO)
Percutaneous coronary intervention (PCI)	First‐in‐human (FIM)
Bioresorbable scaffold (BRS)	

Also, our group has developed a fully bioresorbable PDA occluder. Build on the double umbrella concept, our PDA occluder consists of three parts: an anchoring arm, a stem and an umbrella with spokes with pull‐fold mechanism in order to be repositionable and retrievable during the deployment (Figure 7c). The umbrella serves to cover the PDA and needs to recover as fast as possible postdeployment to ensure a fast delivery procedure. Therefore, PLC is used as the umbrella material for it is elastomeric and possesses good recovery property. The spokes on the umbrella serve as a structural support during deployment as blood pressure turbulence is experienced in the aortic side of the heart. Since both recoverability and mechanical strength are both required for the spokes, the PCL/PLC blend is used to give an optimum mix of recoverability and strength. Similarly, PCL/PLC blend is also chosen as the material for the anchoring arm, which serves to anchor and immobilize the device within the conduit of the PDA. During the sheathing process, the anchoring arm is folded inwards toward the stem to enable the device collapse into the deployment sheath. Once deployed, the four arms should open up and recover the original structure and then rest/grip onto the inner side of the PDA lumen, thus ensuring that the device stays in place. Lastly, the stem serves as the backbone of the PDA occlusion device, connecting both the anchoring arm and the umbrella. This part of the device has to withstand high stress: therefore, the blend of PCL and BaSO4 is chosen as the material for it has the highest modulus among the blends. Furthermore, with the addition of BaSO4 into the stem and anchoring arm parts, the PDA occluder is radiopaque so that it can be easily monitored by fluoroscopy and accurately placed.^61^ Our in vivo feasibility study demonstrated that the PDA device was able to recover within 2–3 min in vivo for immediate PDA closure and tissue overgrowth on the device at 1‐month follow‐up result, indicating the possibility of usage in human being.^62^

## 5 CONCLUDING REMARKS

It is now recognized that opening and closing of selected body vessels or defects is preferably done with a temporary scaffold rather than a biostable one, whose permanence may lead to serious side effects. Our translational efforts in two such fully bioabsorbable cardiovascular implants were seed‐funded by academic grants; we believe these constitute some of the very rare examples of academically driven translation in such implanted devices. Although scale‐up and manufacture of the implants required substantial follow‐on funding, these examples of stents and occluders demonstrate the possibility of pushing inventions in academia to the clinic, slower though the overall process may be in comparison to concerted industrial efforts. The successful translation of our coronary stent was possible because there was significant follow‐on funding available for it, as the coronary stent space is competitive because of its large market size. In contrast, our work on fully absorbable stents for blocked ureteric and trachea, both of which are unmet medical needs at present, remain untranslated because they both address small markets (<$50 M each worldwide). Medical device companies do not pursue such options because the return on investment is not as high as, for example, with coronary stents or ventricular assist devices. The story is similar with occluders: occluder companies are few and far apart, and most if not all are too small to afford R&D in second and third‐generation devices. While our own occluder prototype development was funded largely by academic grants, follow‐on funding was (and remains) difficult to obtain for translating these small‐market devices to the clinic. This is a pity. In the coming years, it is hoped that such efforts, that address genuine medical needs, can be funded by nonprofit organizations or even by academic institutions. Such funding need not be in conflict with University ethics; on the contrary, it may fit in very well with the stated (and unstated) goal of academic institutions to serve their communities in ways beyond education.
